# Is aspartate aminotransferase-to-platelet ratio index a biomarker in the evaluation of advanced fibrosis in non-alcoholic fatty liver disease?

**DOI:** 10.1093/gastro/gou063

**Published:** 2014-11-03

**Authors:** Mehmet Agilli, Fevzi Nuri Aydin, Tuncer Cayci, Yasemin Gulcan Kurt

**Affiliations:** ^1^Department of Biochemistry, Agri Military Hospital, Agri, Turkey, ^2^Department of Biochemistry, Sirnak Military Hospital, Sirnak, Turkey and ^3^Department of Medical Biochemistry, Gulhane Military Medical Academy, Ankara, Turkey

Dear Editor,

We read with great interest the recent article by Tapper *et al.* [1]. The authors aimed to determine the clinical predictors of advanced histology in the referral population. In multivariate logistic regression analyses, they have demonstrated that body mass index (BMI) ≥30 kg/m^2^, female gender, and aspartate aminotransferase (AST) >40 IU/L were associated with a non-alcoholic fatty liver disease (NAFLD) activity score >4. They have also shown that AST-to-platelet ratio index (APRI) >1 was the most significant predictor of advanced fibrosis. Additionally, they have suggested that patients with suspected NAFLD should be routinely evaluated for advanced liver disease, including non-invasive indices of fibrosis such as APRI, and serious consideration given to liver biopsy. Understanding the value of non-invasive indices of fibrosis such as APRI in patients with NAFLD, we consider this large, retrospective study to be very important; however, we have some questions that should be mentioned as contributory factors.

The authors mentioned exclusion criteria: patients with viral hepatitis, alcoholic liver disease, haemochromatosis, Wilson disease, drug-induced liver injury and autoimmune hepatitis were excluded from the study. We wonder whether patients with pancreatobiliary disease and platelet disorders were also excluded from the study. Elevated AST levels might be the result of the pancreatobiliary disease, even in the absence of obvious liver disease [2]. Therefore, amylase, alkaline phosphatase, gamma-glutamyl transferase, bilirubin results and platelet count should also be included in this study.

APRI has an important disadvantage because of the use of upper limits of normal (ULN) for aminotransferases. There is no standardization between laboratories [3]. It was shown that ULN for AST ranged from 26–49 IU/L according to gender, BMI and serum cholesterol in the control population [4]. On the other hand, in another study, it was reported that serum aminotransferase activities were significantly higher in men than in women in a control population [5].

In conclusion, we think APRI results could cause confusing interpretations for the staging of advanced fibrosis in the patients with NAFLD. We need further studies to better understand the relationship between APRI results and fibrosis in patients with NAFLD.
